# P-2108. Risk Factors for Indeterminate Tuberculosis (TB) Testing Results Among Immunocompromised Children in an Area of Low TB Endemicity

**DOI:** 10.1093/ofid/ofae631.2264

**Published:** 2025-01-29

**Authors:** Randal De Souza, Shweta Hegde, Masako Shimamura, Jennifer L Dotson, Kyla Driest, Megan McNicol, Kelly Wise, Christopher Ouellette

**Affiliations:** Nationwide Children's Hospital, Columbus, Ohio; Nationwide Childrens Hospital, Columbus, Ohio; Nationwide Children's Hospital, Columbus, Ohio; Nationwide Children's Hospital, Columbus, Ohio; Nationwide children's hospital, Columbus, Ohio; Nationwide Childrens Hospital, Columbus, Ohio; Nationwide Children’s, Columbus, Ohio; Nationwide Children's Hospital, Columbus, Ohio

## Abstract

**Background:**

Tumor necrosis factor α inhibitors (TNFαi) are immunosuppressants used to treat autoimmune (AI) conditions and inflammatory bowel disease (IBD). TNFαi initiation requires tuberculosis infection (TBI) screening, often performed by interferon-γ release assays (IGRA) including the QuantiFERON Gold Plus (QFT) and T-SPOT.*TB* (T-SPOT). The rate of and risk factors for indeterminate (IND) testing results, as well as the optimal TBI screening assay, among immunocompromised children (ICC) with AI or IBD in an area of low TB endemicity remain poorly defined. We performed a retrospective analysis of QFT testing at our institution to identify rates of and risk factors for IND results. We also prospectively evaluated the performance of QFT versus T-SPOT in an area of low TB endemicity.

**Figure:**
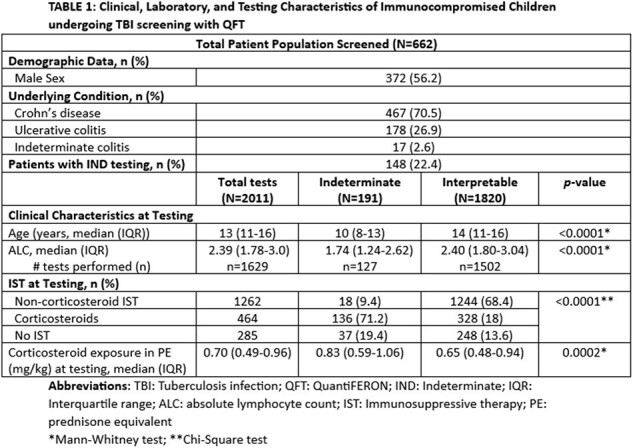

**Methods:**

A retrospective analysis of children (ages 0-21 years) with AI or IBD on immunosuppression cared for at Nationwide Children’s Hospital (NCH) from 1/2010-12/2023 who underwent TBI screening with QFT. Patients were excluded if they had prior TBI, BCG vaccine, and organ or stem cell transplant. Demographic, laboratory results, and medication history were recorded. Prospectively, QFT-eligible patients were consented for concurrent testing utilizing the T-SPOT test, run in parallel. Single variable statistical analysis was performed.

**Figure:**
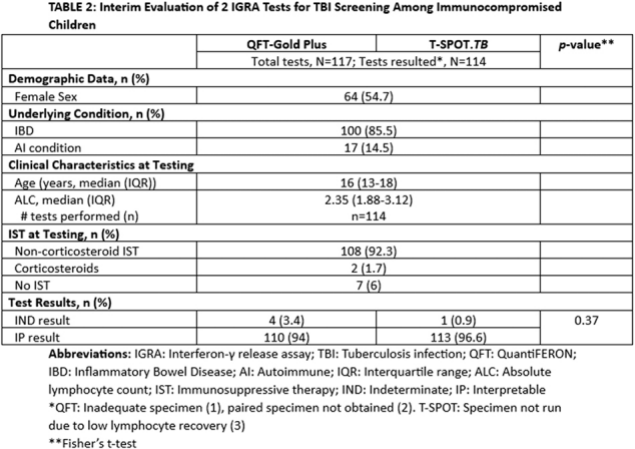

**Results:**

662 patients (372 (56.2%) male) had 2011 unique QFT tests performed. 148 patients (22.4%) had a total 191 (9.5%) IND QFT result at any time in the study interval. Younger age, lower absolute lymphocyte counts (ALC), and corticosteroid exposure at testing were observed more frequently in those with IND QFT results [TABLE 1]. At an interim assessment of the prospective study, 117 patients underwent tandem screening by QFT and T-SPOT; 5 total IND results were observed [QFT (n=4), T-SPOT (n=1)] [TABLE 2].

**Conclusion:**

Younger age, lower ALC, and corticosteroid exposure at time of testing were observed more frequently in those with IND QFT results. While interim assessment of T-SPOT vs. QFT does not yield a difference, this may be driven by a high proportion of individuals with adequate disease control. Ongoing prospective assessment may better define an optimal screening IGRA assay for TBI among ICC with AI or IBD in an area of low TB endemicity.

**Disclosures:**

Jennifer L. Dotson, MD, MPH, Pfizer: Grant/Research Support Christopher Ouellette, MD, Oxford Immunotec: Grant/Research Support|UpToDate: Royalties

